# Estimating the impact of reopening schools on the reproduction number of SARS-CoV-2 in England, using weekly contact survey data

**DOI:** 10.1186/s12916-021-02107-0

**Published:** 2021-09-10

**Authors:** James D. Munday, Christopher I. Jarvis, Amy Gimma, Kerry L. M. Wong, Kevin van Zandvoort, CMMID COVID-19 Working Group, Yang Liu, Joel Hellewell, Nicholas G. Davies, C. Julian Villabona-Arenas, Rosalind M. Eggo, Akira Endo, Nikos I. Bosse, Hamish P. Gibbs, Carl A. B. Pearson, Fiona Yueqian Sun, Mark Jit, Kathleen O’Reilly, Yalda Jafari, Katherine E. Atkins, Naomi R. Waterlow, Alicia Rosello, Yung-Wai Desmond Chan, Anna M. Foss, Billy J. Quilty, Timothy W. Russell, Stefan Flasche, Simon R. Procter, William Waites, Rosanna C. Barnard, Adam J. Kucharski, Thibaut Jombart, Graham Medley, Rachel Lowe, Fabienne Krauer, Damien C. Tully, Kiesha Prem, Jiayao Lei, Oliver Brady, Frank G. Sandmann, Sophie R. Meakin, Kaja Abbas, Gwenan M. Knight, Matthew Quaife, Mihaly Koltai, Sam Abbott, Samuel Clifford, Sebastian Funk, W. John Edmunds

**Affiliations:** Centre for Mathematical Modelling of Infectious Disease, London School of Hygiene and Tropical Medicine, London, UK

**Keywords:** School closure, SARS-CoV-2, COVID-19, Social contacts, Reproduction number, CoMix

## Abstract

**Background:**

Schools were closed in England on 4 January 2021 as part of increased national restrictions to curb transmission of SARS-CoV-2. The UK government reopened schools on 8 March. Although there was evidence of lower individual-level transmission risk amongst children compared to adults, the combined effects of this with increased contact rates in school settings and the resulting impact on the overall transmission rate in the population were not clear.

**Methods:**

We measured social contacts of > 5000 participants weekly from March 2020, including periods when schools were both open and closed, amongst other restrictions. We combined these data with estimates of the susceptibility and infectiousness of children compared with adults to estimate the impact of reopening schools on the reproduction number.

**Results:**

Our analysis indicates that reopening all schools under the same measures as previous periods that combined lockdown with face-to-face schooling would be likely to increase the reproduction number substantially. Assuming a baseline of 0.8, we estimated a likely increase to between 1.0 and 1.5 with the reopening of all schools or to between 0.9 and 1.2 reopening primary or secondary schools alone.

**Conclusion:**

Our results suggest that reopening schools would likely halt the fall in cases observed between January and March 2021 and would risk a return to rising infections, but these estimates relied heavily on the latest estimates or reproduction number and the validity of the susceptibility and infectiousness profiles we used at the time of reopening.

**Supplementary Information:**

The online version contains supplementary material available at 10.1186/s12916-021-02107-0.

## Background

Since the beginning of the COVID-19 pandemic, school closures have been implemented in many countries as part of a broader response to suppress transmission [1]. It is well established that children are at low risk of hospitalisation and death as a direct result of infection [2, 3]. Despite this lower risk, there is concern that allowing transmission amongst younger age groups increases the risk of infection in adults, who are at substantially higher risk. The role of schools in the transmission is therefore an important question. On 4 January 2021, a third national lockdown was announced in England to curb the transmission of SARS-CoV-2 [4]. This included the closure of schools, a measure the UK government reversed on 8 March.

At the time of the decision to reopen, the direct and indirect impact of school closure and reopening was still unclear. To date, there is mixed evidence around the role of schools in community transmission. Existing studies of transmission within schools have wide ranging results [5–7]. Other work demonstrates an increased prevalence amongst school-aged children in the months after schools returned in September 2020 [8, 9] and a higher risk of infections entering households through children than adults. However, studies have failed to find evidence that schools drive transmission in the community [10, 11]. A particular challenge for many observational analyses, based on reported cases, is bias resulting from the age dependence in case ascertainment due to varying rates of asymptomatic infection [12]. This challenge is further complicated by changes in epidemiology due to the emergence of new variants [13].

The potential change in transmission of SARS-CoV-2 upon reopening schools predominantly depends on a combination of two factors: firstly, the age-specific risk of transmission upon contact, and secondly, the likely increased rate of contact between members of the population due to school reopening. Multiple studies aimed at understanding the relative transmission risk associated with children indicate lower susceptibility [14–16] and some indicate lower infectiousness [14]. However, evidence of lower transmission risk amongst children alone is insufficient to quantify the impact of reopening schools. There is a need to combine the estimates of reduced susceptibility and infectiousness with age-specific contact patterns in these age group social contacts amongst school-aged children.

There is abundant evidence that children’s contacts increase when schools are open, presenting opportunities for increased infectious disease transmission which is well documented in other pathogens such as influenza [17]. Nonetheless, it is important to capture how these contacts vary under the specific conditions presented during the current pandemic response, where social distancing and other mitigations are in effect within schools.

Although schools were closed during the third national lockdown, during the second national lockdown (November 2020), restrictions were similar for the majority of the population, but in-person lessons continued in schools. We used data collected as part of the CoMix social contact survey [18] to compare contacts made during these two lockdown periods. We combined these data with estimates of age-stratified susceptibility and infectiousness [14–16] to evaluate the potential impact of reopening schools on the reproduction number in England [14–16].

## Methods

### CoMix data

CoMix is a longitudinal behavioural survey, launched on 24 March 2020. The sample is broadly representative of the UK adult population with data collected from approximately 2000 individuals per week. Participants are invited to respond to the survey once every 2 weeks. We collected weekly data by running two alternating panels. Parents complete the survey on behalf of children (17 years old or younger). Participants record direct, face-to-face contacts made on the previous day, specifying certain characteristics for each contact including the age and sex of the contact, whether contact was physical (skin-to-skin contact), and where the contact occurred (e.g. at home, work, whilst undertaking leisure activities). Further details have been published elsewhere [18]. The contact survey is based on an approach developed for the POLYMOD contact survey [19]. To give insight into the difference in contacts of adults and children between the two periods of lockdown, we include a brief descriptive analysis of the contacts recorded during the November and January lockdown periods by age group and geographical region.

### Constructing contact matrices and estimating reproduction number

Age-stratified contact matrices detail the rate of contact between age groups in the population; they are a central component of well-established methods used for assessing the potential for transmission and analysis of disease dynamics within populations [20]. We constructed age-stratified contact matrices for nine age groups (0–4, 5–11, 12–17, 18–29, 30–39, 40–49, 50–59, 60–69 and 70+ years old). Participants did not report exact ages of contacts; we therefore sampled from the reported age group with a weighting consistent with contacts reported in the POLYMOD survey. We fitted a truncated negative binomial model to calculate the mean contacts between each participant and contact age groups. To ensure reciprocity in contacts, we multiplied the matrix by population size vector for England, using United Nations World Population Prospects data [21], before taking the cross-diagonal mean and then dividing by the same population vector again, which results in the expected rate of contact between age groups in the population surveyed.

### Profiles of age-dependent transmission risk

We considered five age-dependent susceptibility and infectiousness profiles (Table 1).

**Table 1:** Susceptibility and infectiousness profiles taken from Davies et al. [14], ONS reports and Viner et al. [16]

Study	Age groups	Susceptibility	Infectiousness	Clinical fraction
Davies et al.^a^	0–4	0.4 (0.25, 0.57)	0.61	0.29 (0.18, 0.44)
	5–10	0.4 (0.25, 0.57)	0.61	0.29 (0.18, 0.44)
	11–17	0.4 (0.27, 0.53)	0.61	0.21 (0.12, 0.31)
	18–29	0.79 (0.59, 0.96)	0.64	0.27 (0.18, 0.38)
	30–39	0.86 (0.69, 0.98)	0.67	0.33 (0.24, 0.43)
	40–49	0.80 (0.61, 0.96)	0.70	0.40 (0.28, 0.52)
	50–59	0.82 (0.63, 0.97)	0.75	0.49 (0.37, 0.60)
	60–69	0.88 (0.70, 0.99)	0.82	0.63 (0.49, 0.76)
	70+	0.74 (0.56, 0.90)	0.85	0.69 (0.57, 0.82)
ONS^b^	0–4	0.5 (0.35, 0.75)	1.0 (0.7, 1.5)	
	5–10	0.5 (0.35, 0.75)	1.0 (0.7, 1.5)	
	11–17	0.5 (0.35, 0.75)	1.0 (0.7, 1.5)	
	18–29	1.0	1.0	
	30–39	1.0	1.0	
	40–49	1.0	1.0	
	50–59	1.0	1.0	
	60–69	1.0	1.0	
	70+	1.0	1.0	
Viner et al.^c^	0–4	0.64 (0.51, 0.81)	1.0 (assumed)	
	5–10	0.64 (0.51, 0.81)	1.0 (assumed)	
	11–17	0.64 (0.51, 0.81)	1.0 (assumed)	
	18–29	1.0	1.0	
	30–39	1.0	1.0	
	40–49	1.0	1.0	
	50–59	1.0	1.0	
	60–69	1.0	1.0	
	70+	1.0	1.0	
CoMix fit	0–4	0.31 (0.30, 0.31)	1.0	
	5–10	0.31 (0.30, 0.31)	1.0	
	11–17	0.31 (0.30, 0.31)	1.0	
	18–29	1.0	1.0	
	30–39	1.0	1.0	
	40–49	1.0	1.0	
	50–59	1.0	1.0	
	60–69	1.0	1.0	
	70+	1.0	1.0	

^a^95% credible intervals

^b^Approximate results inferred from the plot in [15] unknown quantification of uncertainty

^c^95% confidence interval

The first profile (i) assumed equal susceptibility and infectiousness in all age groups. This is unlikely to reflect reality but provides an upper limit as a reference point to compare the other profiles.

For the second profile, (ii) we used results from a mathematical modelling study by Davies et al. [14] Although this work does not present estimates of infectiousness directly, it does present estimates of relative susceptibility and clinical fraction in 9 age groups. In addition, the work reports an estimate of 50% infectiousness for subclinical cases. We combined clinical fraction with relative susceptibility of sub-clinical cases to calculate infectiousness per age group further detailed in Table 1.

The third profile (iii) was based on the analyses of household transmission patterns from the Office for National Statistics (ONS) Community Infection Study [15]: 50% susceptibility in children relative to adults but equal infectiousness.

For the fourth profile (iv), we performed a meta-analysis of prevalence studies included in a systematic review by Viner et al. [16]. We used a random effects model based on the data from Figure 4 of their paper. This resulted in 64% (51–81%, 95% confidence interval [CI]) susceptibility in children relative to adults; we assumed equal infectiousness between children and adults [16].

For the fifth profile (v), we used an independent estimate of relative susceptibility in children (31%, see The ‘Results’ section), quantified by comparing reproduction numbers estimated from CoMix data and using a well-established time-series method developed by Abbott et al. [22], which uses a time series of cases to determine the instantaneous reproduction number under an assumed generation interval and infection to reporting delay distribution.

### Inferring age dependent transmission risk using CoMix data

We established independent estimates of susceptibility and infectiousness in children relative to adults. We did this by comparing estimates of *R* using CoMix contact data [23] with estimates of the time-varying reproduction number in England calculated using case data [22]. To estimate the infectiousness and susceptibility of children relative to adults, we calculated how change in contact rate as schools returned in September 2020 would affect *R* using two-weekly rolling contact matrices **C**_*t*_ and varying susceptibility and infectiousness profiles by age as vectors **s** and **i**. *R* is given by the spectral radius (largest eigenvalue) of the next-generation matrix, which scales linearly (*r*) with the Hamarand product (∘, element-wise multiplication) of the contact matrix **C**_*t*_ and the outer product (⊗) of the infectiousness and susceptibility vectors:

1 $$ R=r\  Eig\left({\mathbf{C}}_t\circ \left(\mathbf{i}\otimes \mathbf{s}\right)\right) $$

We simplified **s** and **i** such that adult age groups (18+) were 1.0, and child age groups were equal, *s* and *i*. We inferred *s* and *r*, keeping *i* equal to 1.0 for all age groups, by fitting our estimates to those calculated using the EpiNow2 package [22]. We assumed gamma-distributed uncertainty in the time-varying estimates which we parameterised using the mean *μ*_*rt*_ and standard deviation *σ*_*rt*_ of these estimates over each survey period used to calculate CoMix derived eigenvalues.

2 $$ {R}_t\sim \mathrm{Gamma}\left({\mu}_{R_t},{\sigma}_{R_t}\right) $$

We fitted the parameters by maximising the joint log-likelihood (*ll*) of the full sample of estimates of CoMix based *R* estimates to the distribution of time-varying *R* estimates from EpiNow.

3 $$ ll={\sum}_t{\sum}_n\left(\log \left({f}_{R_t}\left({R}_n(t)|{\mu}_{R_t}(t),{\sigma}_{R_t}(t)\right)\right)\right) $$

To show the likelihood surface of relative susceptibility and infectiousness, we calculated the log likelihood (*ll*) of a range of fixed combinations of *i* and *s*, estimating the corresponding value of *r*.

We fit over a period which includes the date when schools reopened, in September 2020, to capture the large change in contacts of school-aged children that coincided with reopening. Schools were closed to the majority of pupils from 23 March 2020 until the new academic year, which began on 4 September. At this time, school children returned to school full time. We chose to fit over the period between 10 June (when the CoMix survey was expanded to under-17-year-olds) and 10 October (when a school holiday commences for a subset of regions in England, complicating contact rates). In addition, there were a number of substantial changes to the survey panel over the summer which interrupted the collection of contacts of school-aged children for 2 weeks in July. We fitted over 2 periods of time within this interval. Firstly, between 27 July and 10 October, which starts after the panel was changed, fitting over this short time also allowed us to most clearly capture the impact of schools returning in the summer whilst minimising issues related to the gradual acquisition of natural immunity and possible seasonal variation in transmissibility. Second, we fitted over a longer period of time incorporating data from 10 June, which provides more data on contact outside of school term, but includes data from a previous survey panel and may be affected more by the gradual acquisition of immunity over the summer months. For the latter, we omitted 2 weeks in July when contacts of children were not recorded.

In both cases, we omitted data at the end of August, due to a short spike in reproduction number estimates, which we understand to be a spurious effect that resulted from large numbers of imported cases from recreational international travel during August. We assessed the sensitivity of the estimates to our choice of the fitted period by fitting to a range of other periods including different combinations of dates between 10 June and 10 October (Additional file 1: Figure S1) as well as some ranges including data until 5 November (when the second national lockdown was called).

### Evaluating the impact of reopening schools on reproduction number

**Fig. 1:**
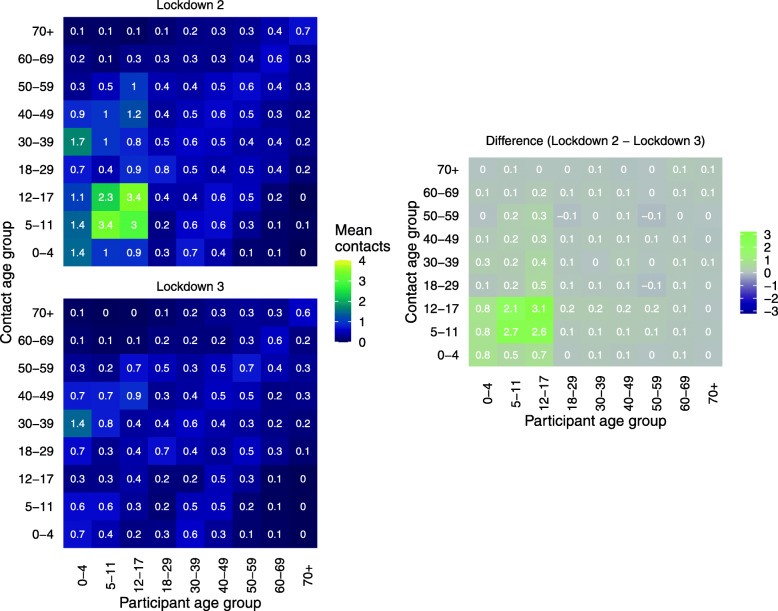
Contact matrix for all contacts in England by age comparing lockdown 2 and lockdown 3 and the absolute difference of the cells of the matrices. Contacts truncated to 50 contacts per participant. Lockdown 2 data from 5 November to 2 December 2020 and lockdown 3 data from 5 to 18 January 2021

We created contact matrices using CoMix data collected during the second lockdown (5 November to 2 December 2020) to represent contacts during a lockdown with schools open. We used data from the third lockdown (5 to 18 January 2021) for contacts during a national lockdown with schools closed (Fig. 1) [23]. We constructed further synthetic contact matrices representing opening primary or secondary schools by replacing the contacts of 5–10-year-olds (primary) and 11–17-year-olds (secondary) in the ‘schools open’ contact matrix (second lockdown), with those from the ‘schools closed’ contact matrix (third lockdown) (Additional file 1: Figure S2).

Since the basic reproduction number scales linearly with the dominant eigenvalue of a matrix of effective contact [20], the ratio of the eigenvalues of two effective contact matrices provides a relative change in reproduction number between the three scenarios considered.

In the case where infectiousness and susceptibility are equal in all age groups, the effective contact matrix is proportional to the contact matrix itself. Under the scenarios where we assumed infectiousness and susceptibility vary with age, we converted measured contact matrices to effective contact matrices by taking the outer product of the estimated age stratified infectiousness profile and susceptibility profile vectors and calculating the eigenvalues of the Hadamard product of the resulting matrix and the contact matrices.

To demonstrate the potential impact of reopening schools, we estimated the relative increase (*k*) in reproduction number (*R*) by calculating the ratio of dominant eigenvalues of the effective contact matrix associated with the respective reopening scenario (**C**_Scenario_) and from the third lockdown period (**C**_LD3_), which was in place between January and March 2021.

4 $$ k=\frac{Eig\left({\mathbf{C}}_{\mathrm{Scenario}}\circ \left(\mathbf{i}\otimes \mathbf{s}\right)\right)}{Eig\left({\mathbf{C}}_{\mathrm{LD}3}\circ \left(\mathbf{i}\otimes \mathbf{s}\right)\right)} $$

We also calculated how *R* varies from baseline values between 0.7 and 1.0, from official UK estimates of the reproduction number from [24].

### Estimating the joint impact of vaccination and school reopening

To capture the scheduled vaccination programme in England and the potential additional immunity it offers, we estimated the expected change in *R* from lockdown restrictions with no immunity from vaccination, to instances with schools open and different vaccine coverage scenarios. We applied these scenarios by modifying the effective contact matrices using vectors of vaccine-derived immunity in each scenario. The vaccination scenarios were based on the UK COVID-19 vaccine strategy; prioritising the elderly and then increasing coverage in younger adults progressively (Table 2).

**Table 2:** Age-specific vaccine-derived immunity used in the three vaccination scenarios we applied as additional susceptibility vectors when calculating the effective contact matrices

Age group	Scenario		
	Vacc. 1	Vacc. 2	Vacc. 3
0–4	0.0	0.0	0.0
5–10	0.0	0.0	0.0
11–17	0.0	0.0	0.0
18–29	0.1	0.1	0.8
30–39	0.1	0.1	0.8
40–49	0.1	0.5	0.8
50–59	0.1	0.8	0.8
60–69	0.5	0.8	0.8
70+	0.8	0.9	0.9

Firstly, to estimate the overall susceptibility by age under each vaccination scenario, we multiplied the susceptibility profiles used in the previous section by the complement of the vaccine coverage in each age group. We recalculated *k* with the modified susceptibility profiles to find the expected increase in *R* as schools open under the respective vaccine scenarios. To adjust these estimates to reflect the total change from a baseline of no vaccination with schools closed, we also estimated the relative change in *R* (*k*_vacc_) as a result of vaccination with schools closed.

5 $$ {k}_{\mathrm{vacc}}=\frac{Eig\left({\mathbf{C}}_{\mathrm{LD}3}\circ \left(\mathbf{i}\otimes \mathbf{s}\right)\right)}{Eig\left({\mathbf{C}}_{\mathrm{LD}3}\circ \left(\mathbf{i}\otimes {\mathbf{s}}_{\mathbf{vacc}}\right)\right)} $$

The overall estimated impact on *R* of the corresponding vaccine-derived immunity and reopening schools is the product of these two factors.

## Results

### Descriptive analysis

**Fig. 2:**
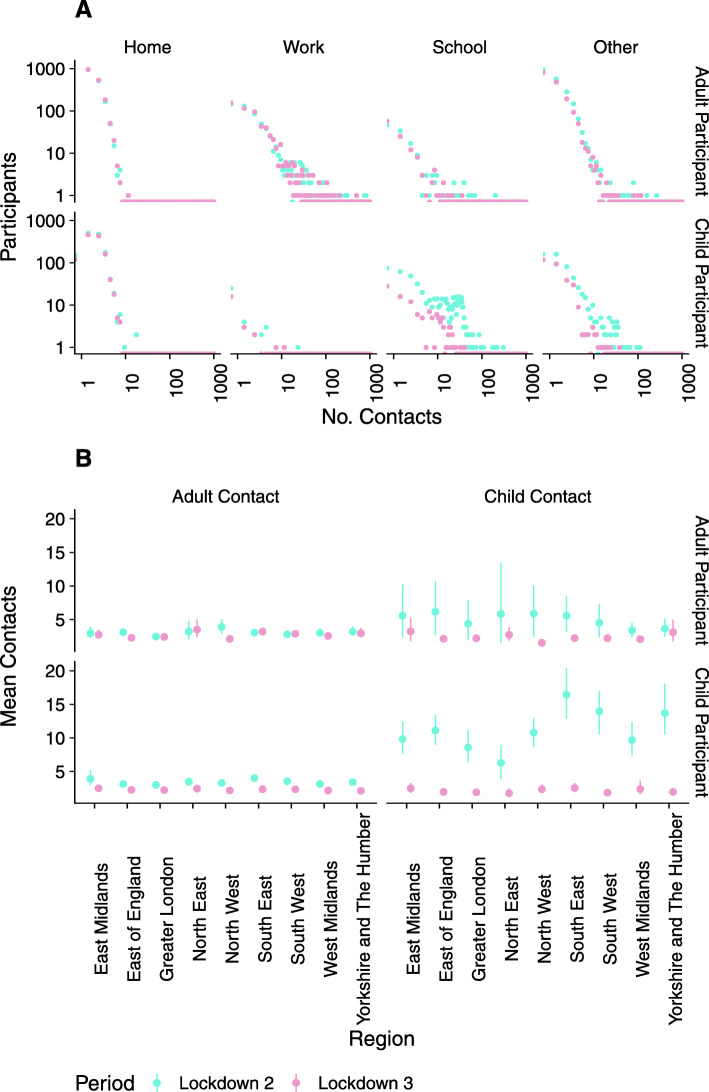
Contacts in the national lockdown periods in November (lockdown 2) and January (lockdown 3). **A** The distribution of the number of reported contacts in home, work, school and other locations for adult (> 17 years old) and child (≤ 17 years old) participants. **B** Mean contacts reported between children and adults in each region of England. Error bars show the 90% CI (bootstrapped, 1000 samples)

Adults’ contacts were similar when comparing both periods of national lockdown; this is consistent across all settings and regions. Although children’s contacts at home were similar between the two periods, contacts at school and ‘other’ locations (contacts that did not occur at home or at school) were consistently higher in lockdown 2 than lockdown 3. Contacts were very similar between lockdowns in all age group combinations other than those between children (Fig. 2). For participants under 18 years old, the mean number of contacts that were also under 18 years old was between 6.3 (3.9–9.0, 90% CI) and 16.7 (13.1–20.4, 90% CI) across the regions of England during the November lockdown. Such contacts were highest in the South East, South West and Yorkshire, and Humber and lowest in London. The mean number of contacts between children reduced to between 1.8 (1.3–2.5, 90% CI) and 2.6 (1.9–3.3, 90% CI) during the third lockdown.

### Estimating susceptibility in children relative to adults using CoMix data

**Fig. 3:**
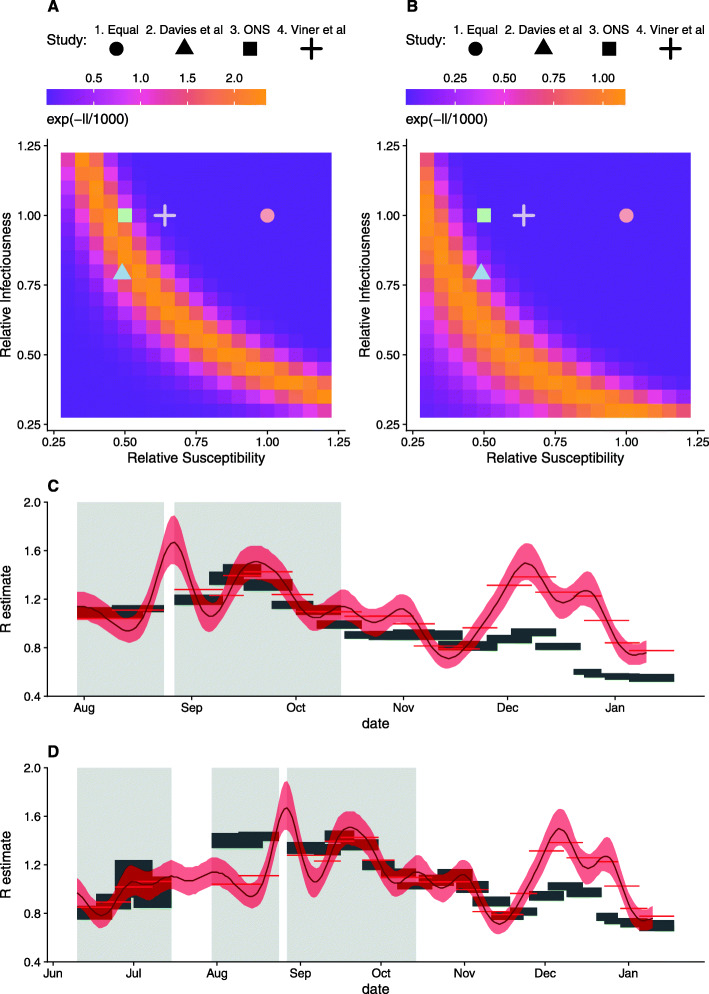
*R* estimates using CoMix data fit to time-varying reproduction number estimates based on the time series of cases [22]. Transformed likelihood for different combinations of relative susceptibility and infectiousness based on data from **A** July to October and **B** June to October and the corresponding *R* estimates in **C** and **D**, respectively. 90% CI of the estimates are shown by grey rectangles for CoMix and the red ribbon for the time-varying reproduction number estimates from case data; red bars show their mean for the CoMix survey periods. Grey-shaded areas indicate fitted periods

Fitting the *R* estimates from CoMix data to time-varying *R* estimates over a period from 27 July to 10 October, we estimated susceptibility of 44% (43.5–0.45.4%, 95% CI) in children relative to adults (Fig. 3A, C), consistent with profiles ii and iii. When we fitted from the 10 June to 10 October 2020, we estimated 31% (29.8–31.4%, 95% CI) relative susceptibility in children compared to adults (Fig. 3B, D), near the lower range of ONS and Davies et al.’s estimates. Although the first estimate corroborates the estimates we used from the literature, the second represents a lower bound on transmission in children; for this reason, we chose to apply the second estimate as the fifth susceptibility profile (v) (Table 1). These results were sensitive to the date range we chose (Additional file 1: Figure S3). Fitting to the periods omitted in the main analysis generally reduces the estimated relative susceptibility, with values between 0.20 and 0.37. However, we believe the data in these periods to be less reliable.

### Evaluation of the impact of reopening schools

**Fig. 4:**
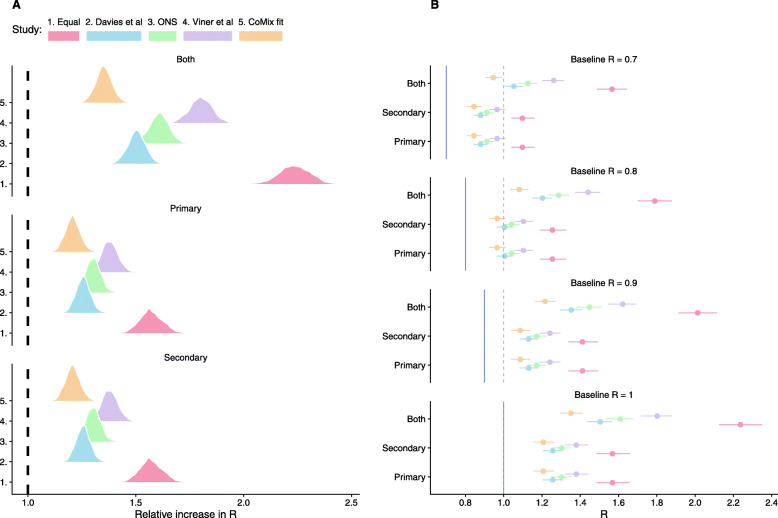
The impact of reopening schools on the reproduction number. **A** The relative increase in *R* (the ratio of dominant eigenvalues between contact matrices for each reopening scenario and that for current contact patterns) under different estimates of the age profile of susceptibility and infectiousness. **B** The estimated *R* after reopening schools (points, 90% CI bars) from baseline *R* of 0.7, 0.8, 0.9 and 1.0 (vertical line). Dashed vertical lines show *R* = 1.0

Incorporating estimates of differential susceptibility and infectiousness of children compared with adults (profiles ii–v), full school reopening increased *R* by a factor of between 1.3 and 1.9 times the baseline value across the four profiles used (including 90% CI range) (Fig. 4, Additional file 1: Table S1). This would result in an increase of *R* from 0.8 to above 1.0 for these four profiles. Partial school reopening resulted in smaller increases in *R* from 0.8 to between 0.9 and 1.2.

When we assumed equal infectiousness and susceptibility between all age groups (profile i), reopening schools resulted in more substantial relative changes in *R*. Full school reopening increased *R* by a factor of between 2.1 and 2.3 (Fig. 4, Additional file 1: Table S1), resulting in an increase of *R* to roughly 1.7–1.9 from a baseline of 0.8 (Additional file 1: Table S1). Partial reopening increased *R* from 0.8 to 1.2–1.3 (Fig. 4).

**Fig. 5:**
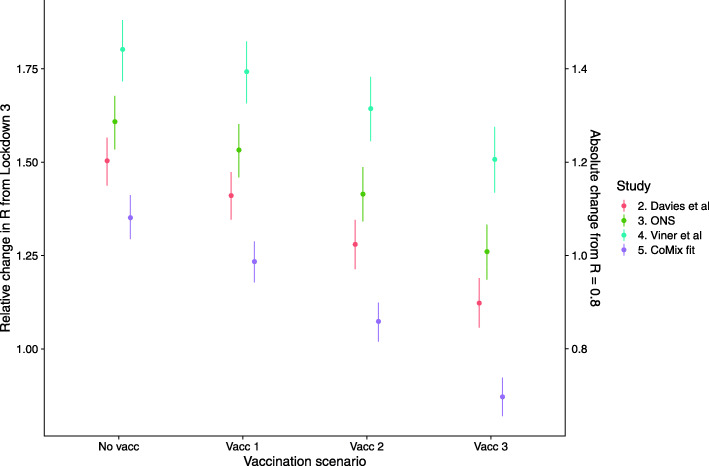
The combined impact of reopening schools on the reproduction number with additional vaccine-derived immunity. The relative increase in *R* (the ratio of dominant eigenvalues between contact matrices for each reopening scenario and that for current contact patterns) for each vaccination scenario, under different estimates of the age profile of susceptibility and infectiousness (colour)

Including possible additional immunity derived from England’s COVID vaccination programme reduced the increase in *R* relative to lockdown 3 (Fig. 5); however, the protective effect of vaccination only outweighed the increase in transmission due to schools when the most extensive vaccination scenario (Vacc. 3) was combined with the lowest estimate of relative susceptibility of children (v, based on CoMix estimates). This resulted in a reduction of *R* by a factor of 0.9 (0.8–0.9, 90% CI). Vaccinating most adults (Vacc. 3) did however result in *R* of 1.0 or below after schools open, from a baseline of *R =* 0.8 during lockdown 3, for three of the scenarios of relative infectiousness and susceptibility (ii., iii and v). However, in the scenario parameterised from Viner et al., *R* remained above 1.0, from the same baseline, for all vaccination scenarios, if schools were opened with *R* increasing to 1.2 (1.1–1.3, 90% CI).

## Discussion

When the UK government made plans to reopen schools on 8 March 2021, the potential impact on the transmission of SARS-CoV-2 was uncertain. Although there have been many attempts to quantify the relative susceptibility and infectiousness of children and adults, these estimates need to be assessed alongside rates of contact to give an indication of the overall risk of transmission in any given setting. We combined social contact data from a large-scale survey in England during two periods of national lockdown, one with schools open and the other with schools closed, with estimates of relative susceptibility of children and adults. We used these data to project the potential impact of reopening schools on reproduction number when schools reopened in March 2021.

Whereas adults’ contacts were generally similar between the two periods of lockdown, there was markedly higher contact between children during the second lockdown (November 2020), when schools were open than the third lockdown (January to March 2021) and when schools were closed. We observed the change in contacts at school but also in other contacts outside of the home. Increased contact outside of school and home settings includes contacts in childcare outside of school hours, which would be expected to rise; however, it could also indicate reduced overall adherence to restrictions amongst children when attending schools physically.

The differences in contacts suggested that reopening all schools would likely increase *R* above 1.0, from an assumed current value of 0.8, if no additional measures (not imposed in the second lockdown) were effective. Reopening primary or secondary is likely to increase *R* above 1.0. This would, in turn, be expected to stop or reverse the fall in cases that had been observed since January 2021 [25]. The risk of cases increasing following the reopening of schools depends greatly on the assumed value of R before schools are reopened. Although cases of the alpha variant (B.1.1.7) appeared to be increasing whilst national lockdown was still in place in November [10, 13], the latest national serology surveys suggest that immunity levels have substantially increased across the UK [25], resultant from both infections and the national COVID-19 vaccination programme. These changes in overall immunity should be reflected in real-time estimates of *R*, but *R* estimates are lagged due to delays in reporting [26]. Although the results vary depending on the estimate of relative susceptibility and infectiousness in children, the qualitative interpretation remains consistent between them. We highlight that we included estimates based on equal infectiousness and susceptibility in all age groups for completeness, but stress that assuming that children are equally infectious and susceptible as adults is not compatible with results from previous studies or our own estimates (Fig. 3).

We found that whilst the increases in *R* due to school reopening would be reduced by additional vaccine-derived immunity, it is unlikely that they would be reversed. We emphasise that these results are indicative only: firstly, because treatment of vaccine-derived immunity is simplistic and based on broad scenarios of vaccine efficacy, and, secondly, because the vaccine-derived immunity profiles we used were likely yet to take some time to materialise. This is due to the pace of the UK vaccine rollout and the delay between immunisation and full immune response, at which time the UK is unlikely to be under lockdown restrictions. Furthermore, it is likely that there was some immunity due to vaccination already in the population prior to 8 March, and therefore reflected in the preopening *R* estimates, this would impact the estimate of both the impact of further vaccine-derived immunity and school reopening.

Our descriptive analysis shows that in November, when schools were open, there was a substantial variation in contacts between children by region. We have not presented regional estimates of the impact of reopening schools on *R*, due to low numbers of observations between the lower-level age group aggregation used in the construction of contact matrices. However, the variation in the mean contacts points to potential geographical variation in the impact of reopening schools, which may be lower in London than in other parts of the country.

Schools have reopened since this work was carried out, providing an opportunity to reflect on how our estimates relate to epidemiological observations around the event of reopening. Based on case reports, it appears that transmission initially remained low allowing cases to continue to fall for a few weeks following reopening [27]. This was however combined with an increase in test positivity amongst school-aged children [28]. This suggests that transmission may have increased in this age group, but those mass testing and quarantine of infectious school children were broadly successful in curbing transmission in schools. We present these suggestions tentatively. As mentioned in the introduction of this paper, evaluation of the impact of reopening is challenging in general. In addition, there are specific events surrounding reopening in March that further complicate evaluation.

In general, real-time estimates of *R* are smoothed substantially by the delay associated with the onset of symptoms and reporting of cases [26]. Since changes in transmission are due to sharp and age-heterogeneous changes in contact, it would be expected that *R* would gradually change as infections reach a new equilibrium age distribution [29]. Also, as mentioned in the introduction, poor case ascertainment in children suggests that detection of the contribution to transmission in schools is likely to be further delayed until this change affects infections in the adult population, which are reported more reliably. This means that sharp changes in contact we expect would lead to a gradual change in *R* making it difficult to associate the changes with a particular event.

More specifically, the reopening of schools in March 2021 coincided with a substantial change in testing, with large-scale home testing being rolled out, particularly amongst school-aged children. This is likely to affect the infection reporting rate. In addition, schools only opened for a short period of time before closing again for the Easter holidays (29 March 2020), where they remained closed for a further 2 weeks.

The combination of these factors makes it difficult to assess whether school outbreaks generally failed to occur due to testing, lower effective contact rates in schools than we anticipated or stochastic variation due to low prevalence in the population.

There are a number of important limitations to this work: Contacts in different settings likely contribute differently to transmission, but we assumed all contacts make equal contributions to transmission, as these differences are not well quantified in the context of control measures. If contacts at school are at lower risk than those outside of school, the impact of reopening schools would be lower. Moreover, contact survey methods are likely to be systematically biassed towards reporting a higher proportion of close contacts than incidental contacts (for example, on public transport); this may lead to an underestimate of the change in contact as restrictions are relaxed, particularly in adults who may be more likely to have this kind of contact. This in turn may overestimate the contribution of contacts of school-aged children to changes in *R* over time. The age-stratified susceptibility profile is likely to change over time as natural immunity is acquired in the population. The profiles we used each reflect a single point in time. Changes in the relative immunity in children would alter the relative impact of school contacts on overall transmission. Changes in the overall immunity over time and seasonal effects on transmission are not expected to affect our main analysis, which presents an instantaneous change in reproduction number under specific contact behaviours. However, there may be some impact on our estimates of relative susceptibility as we fit *R* over a period of time, we suggest that this is likely to be minimal due to the short period over which we fit and the low prevalence of infection during this period. Further, the UK’s testing capacity changed between summer 2020 and spring 2021, although not greatly within these periods. If infections in children were less likely to be identified between July and October 2020 (the period we used to estimate relative susceptibility of children) than they were in March of 2021, we may have underestimated the relative susceptibility when considering the impact on *R* when schools reopened. We counter this limitation by using a wide range of estimates of relative susceptibility in children, all of which give a higher relative susceptibility than our own estimate. We assume adult contacts revert to those observed when all schools were open, which is conservative, in reality, particularly for partial reopening scenarios, adult contacts may not fully return to the same levels. Furthermore, there may also be differences in adherence to restrictions between the two lockdowns, unrelated to school closure. However, the change in adults’ contacts between the two periods was relatively small. The proportion of children in school varied over time due to exclusion-based control measures during the autumn, though the proportion attending school remained high during the November lockdown (Additional file 1: Figure S3). Contacts of children are reported by parents, which may impact their reliability, particularly in school, where parents are unlikely to witness students’ behaviour. The contact survey was conducted in this way for convenience and to allow a quick rollout. We are unaware of any previous work that has established the likely biases that arise from parent-reported contacts. It is unclear whether this would lead to systematic bias in reporting either more or fewer contacts.

Our work evaluates the impact of reopening schools on the reproduction number in England, which gives an indication of how the transmission may be affected by this change. However, there are other factors that reopening schools could introduce, such as the potential for children’s contact at school to provide routes of transmission between households, facilitating long chains of transmission that would be otherwise impossible [30]. We are not able to capture these network effects in this analysis; however, they may play an important role in the change in epidemiology between school closure and reopening. Second, there is evidence for lower prevalence in primary school than secondary schools [8]. Our framework has not captured these differences suggesting there may be additional factors that reduce the impact of reopening primary schools relative to secondary schools. Furthermore, additional management strategies such as mass testing of school children may have served to reduce the risk that a contact in a school results in infection compared to contacts during lockdown 2. Importantly, with the recent emergence of new variants, particularly alpha and delta (B.1.1.7 and B.1.617.2) [31], the baseline *R* will depend on the proportions of these variants as well as contact patterns. Furthermore, these proportions changed substantially over the spring period, likely altering the implications of reopening schools.

Our results suggest reopening schools under the same conditions as November 2020 would have been likely to increase *R* close to or above 1.0, which would stop the decrease in cases observed between January and March. However, precise estimates rely heavily on the baseline values of *R* and the profiles of susceptibility and infectiousness, generally assuming lower susceptibility and no greater infectiousness in children relative to adults. We advocate further evaluation of the impact of within-school measures to assess their contribution to the successful containment of school outbreaks in the weeks following reopening.

## Supplementary Information

**Additional file 1: Table S1.** Expected resultant R if schools were reopened for different baseline values of R, **Figure S1.** Relative susceptibility found by fitting to various parts of the time-varying reproduction number estimate time series. **Figure S2.** Contact matrix for Scenarios included in analysis of school reopening. For all schools open the matrix calculated for Lockdown 2 was used. **Figure S3.** The proportion of child participants who attended school on the day when contacts are recorded.

## Data Availability

Alongside the analysis code, the datasets generated and analysed during this work are available in the GitHub repository located at https://github.com/jdmunday/CoMix_schools_reopening [23].

## Abbreviations

CI: Confidence interval
ONS: Office for National Statistics
UK: United Kingdom
