# Supplementary material for: Estimating the impact of reopening schools on the reproduction number of SARS-CoV-2 in England, using weekly contact survey data
Source: BMC Med. 2021 Sep 10;19:233. doi: 10.1186/s12916-021-02107-0 (PMC8428960; doi:10.1186/s12916-021-02107-0)
Supplement: Supplementary file 1 — Additional file 1: Table S1. Expected resultant R if schools were reopened for different baseline values of R, Figure S1. Relative susceptibility found by fitting to various parts of the time-varying reproduction number estimate time series. Figure S2. Contact matrix for Scenarios included in analysis of school reopening. For all schools open the matrix calculated for Lockdown 2 was used. Figure S3. The proportion of child participants who attended school on the day when contacts are recorded. [file 12916_2021_2107_MOESM1_ESM.docx]

Additional File 1. Supplementary Materials for Estimating the impact of reopening schools on the reproduction number of SARS-CoV-2 in England, using weekly contact survey data

**Table S1** Expected resultant *R* if schools were reopened for different baseline values of *R* reported as median (90% CI)

|  |  | **Baseline *R*** | | | |
| --- | --- | --- | --- | --- | --- |
| **Susceptibility/**  **Infectiousness** | **Attendance** | **0.7** | **0.8** | **0.9** | **1.0**  **(Scale factor)** |
| **1. Equal** | Both | 1.6 (1.5 - 1.6) | 1.8 (1.7 - 1.9) | 2.0 (1.9 - 2.1) | 2.2 (2.1 - 2.3) |
|  | Primary | 1.1 (1.0 - 1.1) | 1.2 (1.2 - 1.3) | 1.4 (1.3 - 1.5) | 1.5 (1.4 - 1.6) |
|  | Secondary | 1.1 (1.0 - 1.2) | 1.3 (1.2 - 1.3) | 1.4 (1.3 - 1.5) | 1.6 (1.5 - 1.7) |
| **2. Davies et al** | Both | 1.1 (1.0 - 1.1) | 1.2 (1.1 - 1.3) | 1.4 (1.3 - 1.4) | 1.5 (1.4 - 1.6) |
|  | Primary | 0.9 (0.8 - 0.9) | 1.0 (0.9 - 1.0) | 1.1 (1.1 - 1.2) | 1.2 (1.2 - 1.3) |
|  | Secondary | 0.9 (0.8 - 0.9) | 1.0 (1.0 - 1.1) | 1.1 (1.1 - 1.2) | 1.3 (1.2 - 1.3) |
| **3. ONS** | Both | 1.1 (1.1 - 1.2) | 1.3 (1.2 - 1.3) | 1.4 (1.4 - 1.5) | 1.6 (1.5 - 1.7) |
|  | Primary | 0.9 (0.8 - 0.9) | 1.0 (1.0 - 1.1) | 1.1 (1.1 - 1.2) | 1.3 (1.2 - 1.3) |
|  | Secondary | 0.9 (0.9 - 1.0) | 1.0 (1.0 - 1.1) | 1.2 (1.1 - 1.2) | 1.3 (1.3 - 1.4) |
| **4. Viner et al** | Both | 1.3 (1.2 - 1.3) | 1.4 (1.4 - 1.5) | 1.6 (1.5 - 1.7) | 1.8 (1.7 - 1.9) |
|  | Primary | 0.9 (0.9 - 1.0) | 1.1 (1.0 - 1.1) | 1.2 (1.1 - 1.3) | 1.3 (1.3 - 1.4) |
|  | Secondary | 1.0 (0.9 - 1.0) | 1.1 (1.1 - 1.2) | 1.2 (1.2 - 1.3) | 1.4 (1.3 - 1.4) |
| **5. CoMix fit** | Both | 0.9 (0.9 - 1.0) | 1.1 (1.0 - 1.1) | 1.2 (1.2 - 1.3) | 1.4 (1.3 - 1.4) |
|  | Primary | 0.8 (0.8 - 0.9) | 0.9 (0.9 - 1.0) | 1.1 (1.0 - 1.1) | 1.2 (1.1 - 1.2) |
|  | Secondary | 0.8 (0.8 - 0.9) | 1.0 (0.9 - 1.0) | 1.1 (1.0 - 1.1) | 1.2 (1.2 - 1.3) |

*
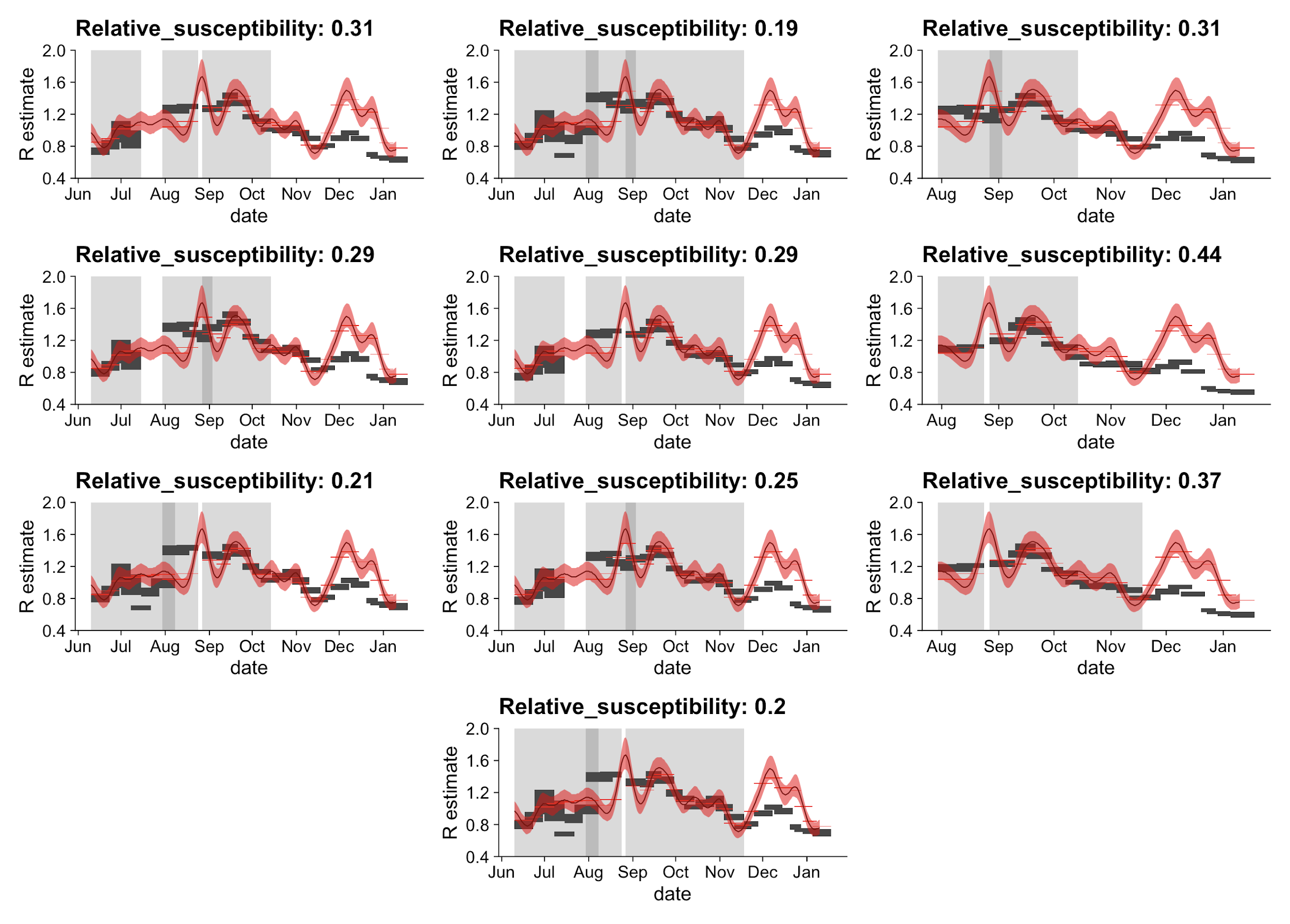
*

***Figure S1: Relative susceptibility found by fitting to various parts of the time-varying reproduction number estimate time series.*** *90% Confidence intervals of the estimates are shown by Grey rectangles for CoMix and the red ribbon for the time-varying reproduction number estimates from case data, red bars show their mean for the CoMix survey periods. Grey shaded areas indicate fitted periods*


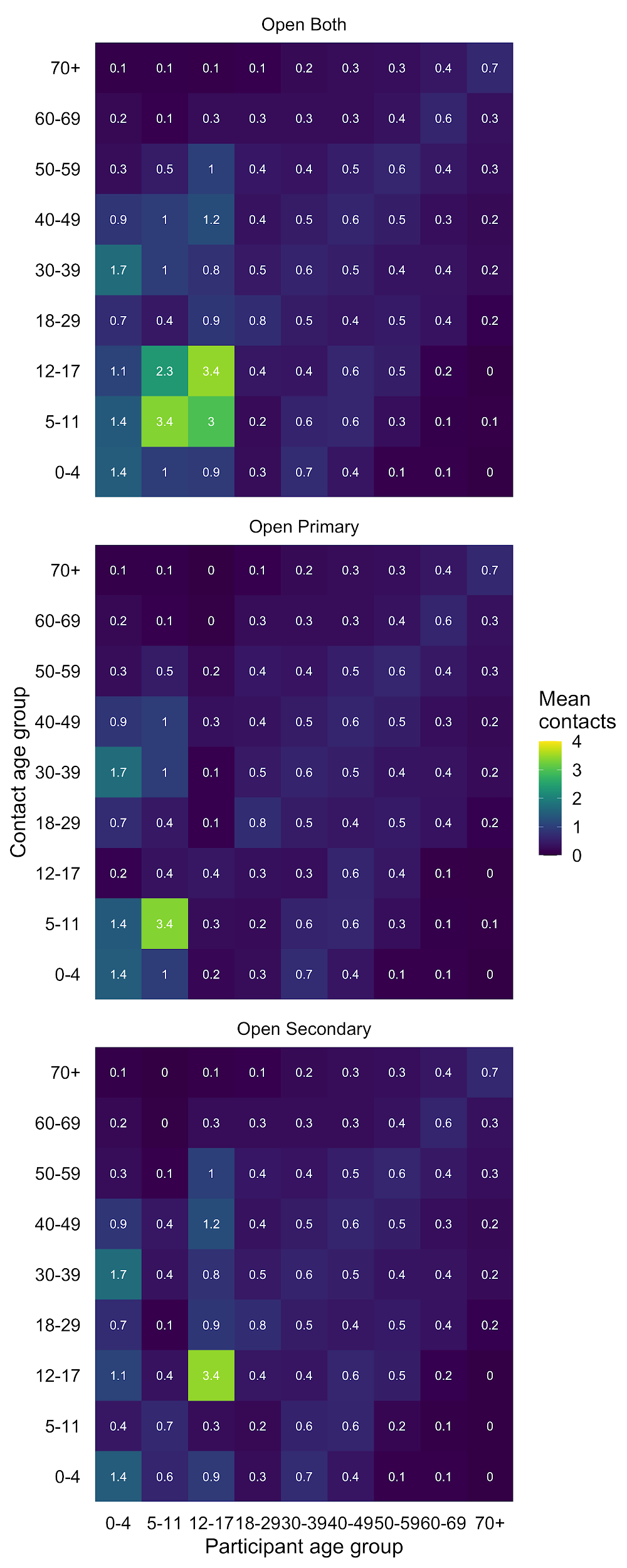


***Figure S2: Contact matrix for Scenarios included in analysis of school reopening. For all schools open the matrix calculated for Lockdown 2 was used.*** *Scenarios with Primary or Secondary schools closed replaced the 5-11 or 12-17 (respectively) column and row replaced with those calculated for Lockdown 3.* Contacts truncated to 50 contacts per participant. Lockdown 2 data from *5th November to 2nd December 2020 and Lockdown 3 data from 5th to 18th of January 2021*

*
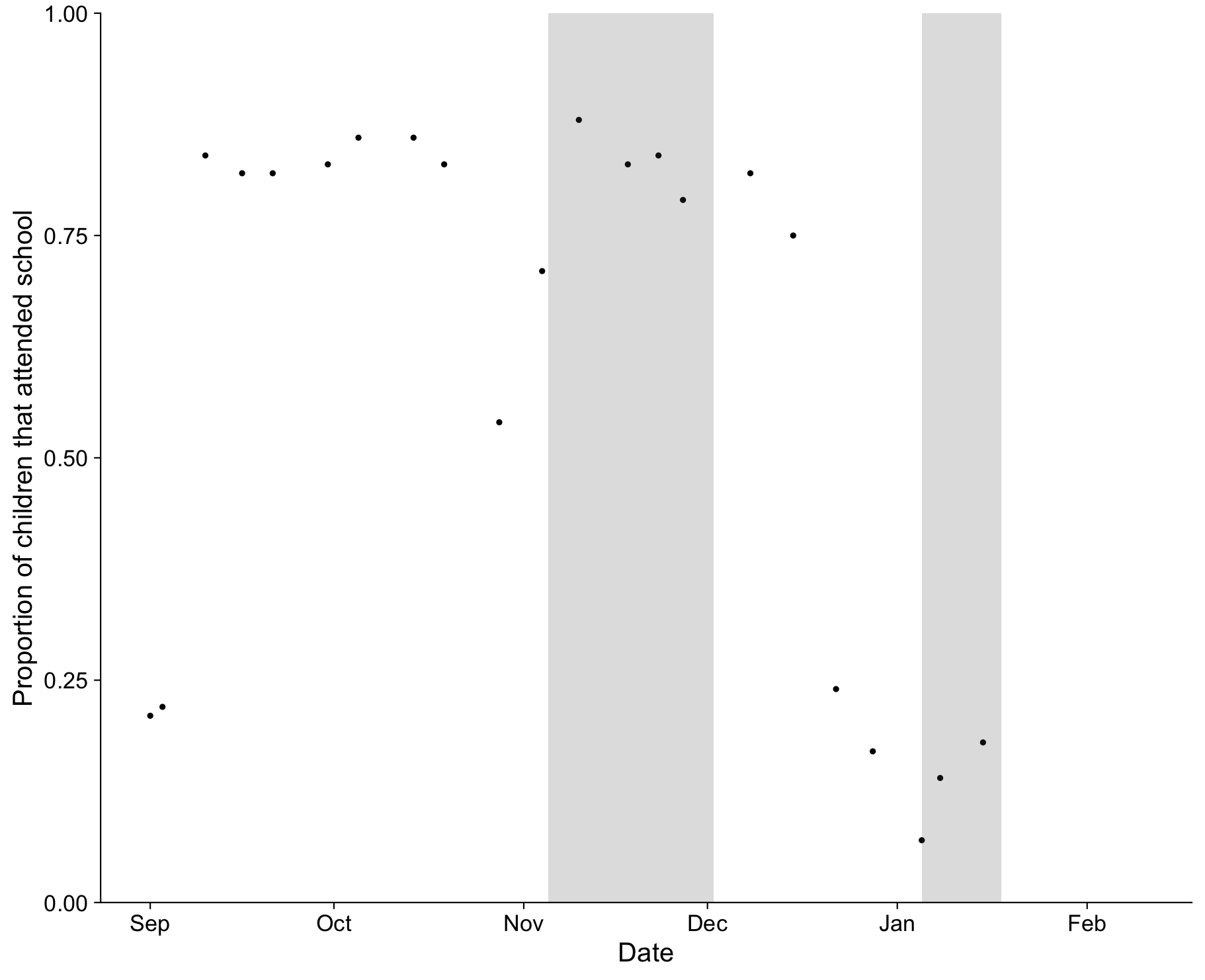
*

***Figure S3: The proportion of child participants who attended school on the day when contacts are recorded (with weekends removed).*** Grey bands represent the periods over which data used for this analysis was recorded (second and third lockdown).
